# Diphtheria and Scarlet Fever

**Published:** 1874-08

**Authors:** 


### DIPHTHERIA AND SCARLET FEVER.

THESE two dangerous maladies are so nearly alike in some of their stages that the real cause of death is not always ascertained until after the fatal termination. Diphtheria very often attacks those who are recovering from scarlet fever; the general causes of both are the same—filth in some form or other in person, clothing, air or habitation; yet two large and wealthy families—one on Murray Hill and the other on Staten Island—have recently been almost exterminated by diphtheria. The one in New York came from the country with half a dozen children in perfect health. They moved into a fine mansion, handsomely furnished; everything, to all appearance, was in unexceptionable order. After the stricken parents had laid four or five of their children in the grave, at intervals of a few days, it was ascertained, on close investigation, that some of the carpets had not been taken up for five or six years. An impressive warning to those who rent "furnished houses."

Noisome cellars often cause both diseases. In New York all the cellars of dwellings open into the interior of the building, with the result of filling every room in the house with the emanations from damp floors, decaying vegetables, and the mustiness attached to any apartment which is made the receptacle for all the cast offs of a family, such as old boots, shoes, hats, umbrellas, stuffed chairs, carpets and the like. There ought to be no cellar to any human habitation ; or, if it must be, the ceiling should be well plastered, and the access should be from the outside. There is not a cellar in the whole city of New Orleans, and taking the year round, the death rate of its resident citizens is less than that of some northern cities, such as New York, Montreal and Quebec ; partly owing, perhaps, to another fact, that no well or spring water is used by the inhabitants, the supply being from tanks above ground, supplied from the roofs of houses.

No well person ought to enter a room where there is diphtheria as a mere visitor, for the fumes will attach to the clothing, especially if woollen, and these communicate disease. Anything coming from the mouth or nose of a diphtheric patient communicates the disease, hence the clothing, handkerchiefs and napkin of the patient should be removed and immersed in some liquid disinfectant, as carbolic acid, say half an ounce of the solid crystals to a gallon of water. While an eminent physician of Baltimore was attending at the bed side of a young lady suffering from diphtheria she sneezed ; he felt a particle enter his nostril ; he was attacked with the disease and died within a week, notwithstanding all was done that was possible by the most eminent medical men of the city. Damp rooms, musty rooms, "spare rooms," unventilated chambers, are made graves for many.

